# Roadmap for maternal behavior research in domestic dogs: lessons from decades of laboratory rodent work

**DOI:** 10.3389/fvets.2024.1394201

**Published:** 2024-06-27

**Authors:** Ming Li

**Affiliations:** Department of Psychology, Nanjing University, Nanjing, China

**Keywords:** maternal behavior, dogs, approach–withdrawal, mesolimbic dopamine, medial preoptic area, prefrontal cortex

## Abstract

Maternal behavior research in laboratory rats has revealed important behavioral and neurobiological mechanisms governing the onset, maintenance and decline of maternal behavior. However, the extent to which these mechanisms are evolutionarily conserved across species is less clear. This manuscript proposes that examining these mechanisms in dogs may be a viable approach to test their generality and help bridge the gap between rodent and human research, as domestic dogs show greater individual differences and exhibit more human-like maternal characteristics than rodents. These aspects represent advantages over rodent models, which in turn allow systems biological approaches not available in rodents. Additionally, domestic dogs share similar social environments with humans, suffer from the same mental disorders as humans, and can be treated with the same medications. This paper begins with a summary of key findings and theoretical developments from decades of rat maternal behavior research, followed by a literature review of the extant maternal behavior research on dogs and related methodology, highlighting the unique behavioral characteristics of dog maternal behavior and similarities and differences from rat maternal behavior. Finally, several knowledge gaps in dog maternal behavior research, as well as the future research in this area is discussed. It concludes that research on dog maternal behavior will not only advance our understanding of the universality of the neurobiological and behavioral mechanisms in maternal behavior, but also improve our understanding of risk factors associated with postpartum mental disorders.

## 1 Introduction

Maternal behavior has always been an important area of research for behavioral neuroscientists, as caring for the young involves multiple interactive physiological systems and brain networks that are altered by reproduction to support postpartum maternal care (the *proximate* mechanisms) (1, 2). Also, maternal behavior entails altruistic behaviors by the mother who sacrifices her own reproductive fitness to ensure the survival and growth of the young, while the young seek to maximize maternal investment. Behavioral ecologists have been interested in this behavior as it allows them to test various hypotheses, such as the parental investment theory and parent-offspring conflict theory to understand how different parenting styles and strategies have evolved and are employed (the *ultimate* mechanisms) (3, 4). Furthermore, because the quality of maternal care has such a profound impact on the brain and behavioral development of the young, this area is intensively studied by developmental psychologists to identify the factors that shape an individual’s personality and mental health (5, 6).

Most behavioral neuroscience studies of maternal behavior studies are carried out in the laboratory rat, an altricial mammalian species that exhibits an elaborated set of behavioral responses that can be easily quantified (7, 8). Decades of research on the behavioral, hormonal and neural mechanisms of rat maternal behavior has provided extensive knowledge about the factors and processes involved in the regulation of different stages of postpartum maternal care (the onset, maintenance and decline) (2, 9–12). One critical issue that has not been adequately addressed is: *to what extent the psychological and neurobiological mechanisms identified in rats can be expanded to other species, including humans*? This issue is important, as it touches on the question of the generality of the maternal mechanisms and may help answer the fundamental question of the origin and evolution of many forms of prosocial behaviors across species (13).

In this paper, I propose that examination of maternal behavior in domestic dogs would allow us to test the generality of basic mechanisms in a way to bridge the gap between research in rodents and that in humans, as it would help reveal the evolved processes of maternal behavior in mammalian species and enhance our understanding of the evolutionary origins and maintenance of cooperation (e.g., alloparental care). Practically, I illustrate how behavioral and neural studies of rat maternal behavior can provide a roadmap to help guide and enhance current research on domestic dogs.

## 2 Significance of dog maternal behavior research

Dogs are a fascinating species of carnivore that either live with humans as pets or live in a social group in free-ranging conditions. Compared to rats, dogs exhibit more human-like maternal care characteristics, such as displaying distinct yet stable maternal caring styles (14) and showing alloparental behavior by male (father) and virgin females (aunt or sister) (15, 16), and even grandmothers (17). Like humans, mother dogs are capable of recognizing their offspring, while offspring can also recognize their mothers, even if they have been permanently separated for 2 years (18). Studying canine maternal behavior is a great way to gain insights into behavioral and social controls of human parental behavior. Because of their relatively larger brain sizes and their willingness to work with humans, they can be subjected to functional Magnetic Resonance Imaging (fMRI) and Positron Emission Tomography (PET) brain imaging analyses under the awake condition while their responses to infant cues can be presented, allowing us to conduct systems neuroscientific investigations to identify the brain mechanisms involved in the sensory perception, maternal motivation, emotional attachment and learning and memory in mother dogs (19). Findings from dog brain imaging work can then be compared and contrasted with those from rat imaging studies (20) and human brain imaging studies (21–26). Because rats do not have convoluted neocortices, studies on the brain of dog maternal behavior could bridge the gap between rats and humans.

Also, dogs’ behaviors are heavily influenced by human factors due to their strong bonding with humans and their co-evolution with humans (27). Dog maternal behavior provides a window through which we can study how human social factors (such as [dog-raising] culture and language used to interact with dogs), as well as human environmental factors influence maternal behavior. Additionally, like humans, some dogs suffer from major depression, anxiety, and aging-induced dementia (28), and they are often treated with the same medications prescribed for humans. Therefore, studying maternal behavior in domestic dogs, as opposed to in rats, might be possible to gain better insights into the etiology of human mental disorders and test the efficacy of new treatments.

Another advantage of dog maternal research is that compared to humans, dogs have a shorter postpartum period (<12 weeks) with known living conditions, genetic backgrounds and life histories. Thus, longitudinal and mechanistic studies on what causes the changes of maternal behavior across postpartum become more feasible, especially on the late decline phase, an area severely understudied (12). Tracking changes in maternal behavior throughout the postpartum period also allows us to investigate how reproductive experiences alter psychological and brain functions involved in maternal care. This work has significant clinical implications, as reproductive experiences are known to influence cognitive, emotional and brain functions of human mothers and may contribute to sex differences in cognitive decline (29, 30). Finally, studying dog maternal behavior also has practical significance. In order to produce successful service dogs through selective breeding, maternal behavior, such as nursing styles, is an important factor to consider (31). For example, Bray et al. (32) found that mother dogs whose nursing style required greater effort by puppies (e.g., mother sitting or standing position) were more likely to produce successful offspring in a guide dog training program, whereas those whose nursing style required less effort (e.g., mother lying on stomach) were more likely to produce puppies that failed. Therefore, being able to identify the maternal nursing style of mother dogs early on or even change their nursing styles would greatly improve the success rate and save a tremendous amount of resources (33), considering that service dogs often require extensive training and cost up to $50,000 (27) with only an approximately 70% of success rate, even in those who are being bred and raised with the specific aim of becoming guide dogs (32). For military working dogs or police dogs, the success rate is even worse (approximately 30%) (31). Furthermore, if we could determine how different maternal caring styles influence psychological traits of offspring (e.g., attention, inhibitory control, problem solving, etc.), it would greatly benefit dog owners when they decide what kind of dog puppies to adopt, allowing better and more compatible human–dog relationships to be built and fewer incidences of later abandonment. Research has also shown that when a puppy dog is being separated from its litter and dam for adoption can greatly influence its later owner-reported undesirable behaviors (34). Many social and behavioral deficits observed in adult dogs may be affected by separating puppies too early from the dam and littermates (6 weeks vs. 12 weeks) (35). Thus, studying dog maternal behavior and identifying various factors that could shape the behavioral development of the young can also help raise more human-friendly dogs for adoption (36). Altogether, dog maternal behavior is situated at a niche position, allowing us to address some of the fundamental scientific, clinical, and practical questions.

## 3 Overall organization of the current manuscript

This manuscript is organized in the following fashion. First, I briefly summarize several key empirical findings and theoretical frameworks from decades of rat maternal behavior research, highlighting research on its regulation, ontogeny, behavioral and neurobiological mechanisms. Next, following the exact same organization of my summary, I review the status of current research on dog maternal behavior, focusing on the methodology and its unique behavioral characteristics. Limited research on the endocrine mechanisms involved in dog maternal behavior will also be discussed and compared to rat maternal work. I conclude that research on dog maternal behavior will benefit greatly from learning rat maternal behavior traditions.

This paper is selective in terms of the topics and studies it describes and in terms of the topics it does not cover. For example, I do not discuss many important studies on mice (37–41), despite the fact that the mouse has become the preferred model system for the study of the neurocircuit mechanisms of parental behavior in recent decades (42, 43). This narrow approach should not be construed as a denial of important work on mouse maternal behavior. It simply reflects my opinion that empirical and theoretical knowledge gained from decades of rat maternal behavior research could provide an adequate “roadmap” for future dog work. In addition, I do not discuss how maternal caregiving experience alters psychological functions in mothers, such as reduced anxiety, enhanced spatial learning and memory, lack of sexual behavior due to lactational anesthesia, or increased feeding behavior (44), as these are the consequences of maternal care, not the mechanisms governing its appearance and expression. I also do not discuss the genetic or epigenetic mechanisms that might mediate maternal behavior (45). Finally, I do not cover the very important developmental impacts of maternal care on the behavioral and neural development of infants (46, 47).

## 4 Rat maternal behavior: a brief overview

Maternal behavior in rats is highly motivated and well-organized social behavior. Under the natural and normal condition, a female rat shows the full range of maternal behavior as soon as she gives birth. Parturition usually occurs on Day 22–23 gestation. During parturition, the mother rat rigorously licks the vaginal opening and helps the pups as they emerge from the birth canal. Once the pups come out, the dam tears off the membranes surrounding the fetuses, eats the placenta and licks the pups. At the end of the parturition, she reconstructs the nest, retrieves the displaced pups and gathers all the pups together in the nest site, and adopts a nursing posture over the pups (7, 48). During late gestation, the dam develops maternal aggression toward intruders and becomes most aggressive between days 1 and 5 postpartum (49). Thus, four principal components of rat maternal behavior are commonly quantified as behavioral measures of maternal behavior: pup nursing or crouching over the young, pup retrieval, pup licking/grooming, and nest-building (8). Rat nursing postures include high crouching (kyphosis), low crouching, and passive or prone nursing (50). Pup retrieval occurs when a pup moves itself or is being removed from the nest. Pup licking is divided into anogenital licking and whole body (general) licking. Nest building involves pushing and carrying nesting materials towards a nest site. Finally, maternal aggression involves aggression against a male intruder. It has also been used as an indirect way of examining the sensory, hormonal, and neural control of maternal behavior (51).

Research over the past 70 years has produced a variety of behavioral testing tools to assess different components of maternal behavior in rats, as well as the underlying psychological functions, including reward processing (52–55), emotional states (56, 57), learning and memory (58, 59), and executive functions (54–56, 60) that are necessary to carry out temporally and spatially appropriate maternal responses.

## 5 Key findings and theories from rat maternal behavior research

### 5.1 Maternal behavior is regulated by a multitude of internal and external factors

The internal factors consist of the hormonal, neuronal and psychological changes in the mother from pregnancy to early postpartum. Rosenblatt and Lehrman (7) refer to this internal source of signals as “maternal condition”: “*changes in the mother’s physiological condition of readiness to perform maternal responses*” (page 43). The effective hormonal profile that stimulates the onset of maternal care is the high levels of estradiol, oxytocin and prolactin superimposed on a background of progesterone withdrawal in early postpartum (10, 61, 62).

The external signals mainly reflect pups’ physical characteristics and psychological traits, including their sensory (e.g., skin color, olfactory cues, body temperature, ultrasonic vocalizations, etc.) and motoric (e.g., motionless, crawling, running, etc.) characteristics and their emotional states [e.g., sick, hungry, or separated from dams (63–68)]. Certain features, such as visual (e.g., the appearance of fur), auditory (e.g., decreased ultrasonic vocalization calls), olfactory and tactile stimulation provided by the pups to the mother and their motor activities play important roles in maternal behavior across postpartum. In addition, specific environments (e.g., home cage, open field, or elevated plus maze; in the presence of a male intruder) also provide external signals to influence the expression of maternal behavior.

### 5.2 Maternal behavior can be induced in male, juvenile, and virgin female rats

Males, post-weaning juveniles (both sexes), and virgin female rats do not spontaneously display caregiving behaviors towards pups, often actively avoid them, indicating that there is an avoidance/withdrawal motivation system that prevents them from being maternal instantaneously. This system serves an evolutionary purpose (i.e., the genetic relatedness of pups to non-mothers is uncertain). However, in the laboratory setting, through continuous exposure to pups, these non-mother rats can overcome this inhibition and become maternal, a process termed “pup sensitization.” The length of sensitization can range from 1 (e.g., 20 days old juvenile females) to 7 days (adult males) and shows clear sex and age differences (69, 70). The phenomenon is an important piece of evidence suggesting the expression of maternal behavior is independent of hormonal controls and the basic neural systems are present in all members of the same species and throughout development from the prepubertal period into older age.

### 5.3 Interaction with pups induces maternal memory that maintains a long-term enhanced level of maternal responsiveness

The maintenance of maternal behavior after parturition and throughout the remainder of the lactational period prior to weaning depends on prior maternal experience (71, 72). Newly parturient rats need only a few hours of interaction with pups to maintain a high level of maternal behavior. This retention of maternal responsiveness can be revealed at a later test point (e.g., 25 days following the removal of the litter) when pup experienced dams are compared with non-pup experienced dams (71, 73, 74). The same experience effect could be induced in the virgin female rats (74). This long-term maintenance of maternal responsiveness as a function of experience has been termed the *maternal experience effect* or *maternal memory*. Apparently, an additional function of maternal experience effect is to dampen the initial avoidance/withdrawal responses found in non-mothers. The formation of maternal memory depends on protein synthesis (75) and activation of various neurochemical systems, including oxytocin (76), opioid (77), β-noradrenergic receptor (78) and dopamine D_1_ and D_2_ receptors (79, 80). The nucleus accumbens, especially its shell subregion, plays a critical role for the consolidation of maternal memory (58, 59).

### 5.4 Two opposing motivational processes (approach and withdrawal) mediate the onset of maternal behavior in rats: the approach–withdrawal model and its implications in the decline of maternal behavior towards weaning

One of the prominent conceptual frameworks in maternal behavior is the Approach–Withdrawal model (81). This model proposed that there exist two opposing motivational processes (approach and withdrawal) that govern an adult’s responses towards pups. Upon being stimulated by pup stimuli, both processes are activated simultaneously but often not equally. For non-mother rats (e.g., males and virgin females), the withdrawal system is stronger than the approach, resulting in initial avoidance and withdrawal responses towards pups. After parturition and in early postpartum, the approach motivation gains more strength while the withdrawal motivation is being suppressed, mainly due to the impacts of parturitional hormones, leading to the display of maternal behavior.

Li (12) recently extended the Approach–Withdrawal motivational model to account for the maintenance and decline phases of maternal behavior. This extended model suggests that maternal behavior across the onset, maintenance and decline phases all reflects the dynamic competition between these two opposing motivational systems. The onset of maternal behavior occurs when the approach responding to pup stimuli and engaging in maternal behavior becomes stronger than the avoidance or withdrawal responses towards such stimuli. Maternal care is maintained due to the slightly stronger approach system over the withdrawal system. Maternal behavior decline is initiated when the withdrawal system dominants and overpowers the approach system. In late postpartum, mother rats are observed to show more pup withdrawal and rejection responses, such as kicking and pushing pups, pinning them to the floor, or lifting them in the mouth and tossing them aside. Sometimes the females would climb to the top of the feeder to avoid the pups (82).

### 5.5 The approach and withdrawal motivational systems are mediated by two interactive and competing neural systems (i.e., the excitatory system and inhibitory system)

One exciting finding that provides strong support for the Approach–Withdrawal motivational model is the identification of the two antagonistic neural systems mediating the maternal *Approach* and *Withdrawal* motivation, respectively (1, 2, 81, 83, 84). The *excitatory* system, which centers around the hypothalamic medial preoptic area (MPOA) and the adjoining ventral bed nucleus of the stria terminalis (vBNST), and their reciprocal projections to the nucleus accumbens (NAc), ventral tegmental area (VTA), paraventricular hypothalamic nucleus (PVN), basomedial and basolateral amygdala (BMA/BLA), dorsal raphe nucleus (DRN), and periaqueductal gray (PAG) supports the maternal *approach* motivation (42, 59, 85–90). In contrast, the *inhibitory* system, which consists of the main olfactory bulbs (MOB) and accessory olfactory bulbs (AOB), medial amygdala (MeA), anterior hypothalamic nucleus (AHN), dorsomedial and ventromedial hypothalamus (DMH/VMH) and their projections to PAG, mediates the avoidance and withdrawal response (2, 91–96). As expected, functional disturbance of any component of the excitatory system tends to cause maternal disruptions, often reflecting a deficit in maternal motivation (97–101), whereas disturbance of any component of the inhibitory system leads to maternal enhancement, especially in inexperienced animals (e.g., virgin female and male rats) (102–105). They also have multiple contacting points that allow one system to influence another (1, 2, 106). One prominent pathway is the MeA-to-MPOA pathway (104).

### 5.6 The prefrontal cortex is hypothesized to regulate changes of maternal behavior across postpartum by interacting with the neural excitatory and inhibitory systems: a triadic model

Although the Approach–Withdrawal model appears to be adequate in explaining the onset, maintenance and decline of maternal behavior, it does not address *how the two competing systems are coordinated*. Li (107) proposes a triadic model, emphasizing the regulatory role of the medial prefrontal cortex (mPFC) on the Approach and Withdrawal neural systems in different stages of the maternal behavior cycle. Based on the extensive evidence implicating this region in executive functions (108–112), its interconnections with numerous cortical and subcortical brain regions within the maternal brain networks (90), and the fact that various neurochemicals, both those “general synchronizers” (e.g., dopamine, serotonin, etc.) and “specific coders” (e.g., oxytocin) for maternal behavior (113) are found in the mPFC to modulate maternal behavioral functions (54, 114, 115), Li (107) suggests that the mPFC may interact with both the excitatory and inhibitory maternal neural networks to exert an executive control of different patterned maternal responses across the postpartum. The mPFC may exert its regulation of maternal behavior by orchestrating several distinct psychological processes (e.g., neophobia, affective and motivational processing, maternal memory, executive function, etc.) that are critical for the onset, maintenance and decline of maternal behavior.

## 6 Maternal behavior in dogs: basic behavioral characteristics and methodology

There are over 400 breeds of domestic dogs with diverse morphology and functions (116). Despite some differences (14), the basic maternal behavior across different breeds is quite similar, consisting of nest-building, anogenital and whole body licking of the newborn, nursing and direct contact with puppies, and aggression against intruders (117), all of which are also typical behavioral responses of mother rats. This is not surprising given that dogs and rats are altricial species with newborns mainly deaf, blind, and immobile and rely on dams to provide nutrition, warmth and protection. A typical gestation lasts approximately two months (~62–65 days), followed by 3–12 h of parturition (whelping), and another 2–3 months of postpartum. The average litter sizes range from 1 to 12, with an average of 5–6 puppies (118). Different breeds of dogs do exhibit differences in maternal caring styles, thus, studying the breed differences could gain a deeper understanding of the genetic basis of maternal behavior and how maternal behavior is shaped by human selective breeding.

Like rats, nest building in dogs appears about 1 week before whelping, while other components of maternal behavior appear immediately after whelping (119). During parturition, once a puppy comes out, the mother breaks the fetal membranes and licks the newborn’s head and mouth, helping with respiration. The time gap between each puppy can range from a few minutes to 1.5 h. At the end of the parturition, she gathers all puppies together and adopts a nursing posture over them. In the first postpartum week, feeding is initiated by the mother dog who licks puppies’ backs to wake them up and pushes the newborns towards the nipples. There are at least three nursing postures: lateral nursing (mother lying on side), vertical nursing (mother sitting/standing), and ventral nursing (mother lying on stomach). Differences in these nursing postures play an important role in the cognitive and temperament development of puppies (14, 32).

In free-ranging condition, mother dogs are observed to spend the most time with their puppies in dens and nursing occurs for almost the entire observation period (e.g., 30 min) during the first 2 weeks postnatally. They occasionally venture out of their dens for a short time to collect food (16), but this short foraging trips become more frequent from the third week onwards. Free-ranging mother dogs nurse their pups until the pups reach 11–13 weeks of age. Starting in the 4th week of postpartum until the end of 9th week, lactating mother dogs also are observed to provide their biological and kin-related puppies with food through food regurgitation (i.e., disgorging the partly-digested food) (16), termed “epimeletic vomiting” (120). This behavior, considered as “prolongation of the lactation activity” (121), appears more frequently after three weeks postnatally, coinciding with puppies’ increased interest in solid food, and can last up to 5 months postnatally. It is elicited by puppies’ behaviors towards mother dogs, including a variety of movements and gestures as well as a specific type of vocalization (121, 122). Unlike the clearly defined 3 to 4 weeks of the rat postpartum period, the dog postpartum nursing period has not been determined, but can last up to 10 to 13 weeks (118). With the progress of postpartum period, the duration and frequency of nursing, licking and staying close to puppies decline, while time spent away from puppies increases (123). At around 3 weeks of age, puppies begin to leave the nest box and can defecate and urinate on their own and can be weaned as early as 3 to 4 weeks of age, although most puppies are fully weaned to solid food between 7 and 10 weeks of age. Pierantoni et al. (34) found that compared with dogs that remained with their social group for 60 days, dogs that had been separated from the litter earlier (30 and 40 days) were more likely to exhibit potentially problematic behaviors (e.g., destructiveness, excessive barking, fearfulness on walks, reactivity to noises, toy possessiveness, food possessiveness and attention-seeking, etc.).

Maternal caregiving behaviors serve the same functions in both rats and dogs to ensure the survival and development of the young. For example, anogenital licking during the first 3 weeks of life helps puppies urinate and defecate (124), as seen in rats (125). In rats, mothers lick male pups more than females (126), however, whether the same happens in dogs is not known. Nursing behavior in both dogs and rats not only provides milk, but also serves to warm the young who lack the thermoregulatory ability in the first week after birth (127, 128). Maternal aggression in both species protects the young from possible injury by intruding animals.

Besides these shared maternal functions (e.g., feeding the young, keeping them clean and warm, protecting them from intruders) between rats and dogs, a mother dog is unique in that she also “educates” the young, especially in the free-ranging condition. When the puppies are about 8 to 10 weeks old and ready to be weaned, a mother dog is observed to exhibit growling, snarling, muzzle grasping and pacifying behaviors (e.g., licking own lips, licking and pawing, etc.) to teach puppies how to behave in a social group (129).

Research on dog maternal behavior, especially in the laboratory and home settings, is routinely conducted through video recording and offline coding. Focal behaviors representing the main components of dog maternal behavior, including nursing, anogenital and whole-body licking, and staying close to puppies are easily quantified in terms of the duration and frequency. Santos et al. (130) provided a nice summary of the current research methods. One common method mentioned in (130) is to record maternal behavior daily or on selected days for 10 to 15 min in the morning and/or afternoon, 1 to 4 sessions per day in the early postpartum weeks (weeks 1 to 3). The overall goal is to capture the periods when maternal behavior is most likely to occur (e.g., when mother dogs return to the whelping boxes after a walk). It should be noted that although short recording time (<1 h) may be able to capture the majority of maternal responses (e.g., dam presence, nursing, etc.), it may miss low-frequency behaviors, such as anogenital licking. In this case, a longer recording or continuous recording is needed (131).

For free-ranging dogs, behavioral sampling throughout a pre-determined observation period (e.g., from gestation, parturition to 12 weeks postpartum) is often used. This method allows for a direct observation of spontaneous maternal behavior in dogs and has the advantage of observing maternal care exhibited by members of a pack other than the mother. For example, Paul and Bhadra (15) followed 23 litters belonging to 15 dog groups over a period of 15 weeks, starting on the 3rd week until the 17th week postpartum. They observed caregiving behaviors towards puppies from adult dogs (males and females) for two morning (0900–1200 h) and two evening (1400–1700 h) sessions over a 2 week period. In each 3 h observation session, they did 18 1 min instantaneous scans of any behavior that was shown by an adult towards a puppy, and 18 5 min all occurrences sessions.

## 7 Commonalities and differences between rat and dog maternal behavior

### 7.1 In a general sense, dog and rat maternal behaviors are regulated by the same sets of internal and external factors, including pup characteristics and maternal conditions

Parturitional hormones are major internal factors that control the onset and expression of maternal behavior. In dogs, changes in estrogen, progesterone, prolactin, and oxytocin from pregnancy, around parturition and throughout the lactation period are likely to act upon the brain to promote maternal care (132), similar to their roles in rats (133, 134). Indeed, similar changes in these hormones as reported in other species (e.g., sheep and rats) are also found in dogs (135). For example, serum progesterone falls precipitously prior to parturition and remains low during early postpartum. In the meantime, serum levels of cortisol and prolactin peak a few hours prior to parturition, then are subsequently reduced a few hours after birth, but remain elevated during lactation (135). After parturition, how pup stimulation, such as suckling, or maternal caregiving behavior, affects the levels of these hormones has not been studied. Thus, the specific patterns of hormonal changes important for the onset and expression of maternal behavior in dogs have not been determined. This is because most studies only measure levels of these hormones without conducting any mechanistic studies (e.g., manipulating levels of hormones and see how it affects maternal behavior). Ogi et al. (136) even failed to find a positive correlation between salivary oxytocin and maternal behavior in 25 lactating Labrador Retriever dogs. This negative finding may be due the fact that salivary oxytocin does not necessarily reflect central oxytocin function (137), and other methodological issues (e.g., stress) may have masked this relation. Kockaya et al. identified a correlation between the low serum level of oxytocin and maternal cannibalism (i.e., a dam consumes her offspring) in mature female Kangal dogs (132). They found that serum oxytocin was significantly lower in dogs with a previous history of maternal cannibalism, indirectly suggesting that oxytocin might be an important factor contributing to the normal onset of maternal behavior in dogs (132). Apparently, more studies need to be conducted to elucidate the neuroendocrine control of maternal behavior in dogs.

One “maternal condition” that influences maternal behavior is maternal experience (parity and practice). Although in early postpartum multiparous rats appear to exhibit an enhanced maternal responsiveness, the overall difference between primiparous and multiparous rats is small. Both perform at the comparable level and show a similar decline in late postpartum (138). Also, both groups show a similar level of improvement in pup retrieval efficiency with repeated testing (139). In dogs, a clear parity and practice effect has been reported (140). Primiparous mothers lick puppies more than multiparous mothers and show a progressive increase in contact with, licking and nursing puppy throughout the first 3 week postpartum. In contrast, multiparous mothers tend to have a consistent and higher physical contact time, nursing and licking throughout than primiparous mothers (140).

The limited research on the influences of pup characteristics on dog maternal behavior indicates some similar results as found in rats (141, 142) and sheep (143). For example, the importance of amniotic fluid in initial maternal acceptance in those species has also been demonstrated in dogs. Abitbol and Inglis (144) found that new mother dogs would not accept puppies if they had been washed three times so that the odor of amniotic fluid was removed. However, they would accept washed puppies if they were bathed in their amniotic fluid and the placenta and membranes were returned to the mothers. This study indicates that the odor of amniotic fluid is a potent stimulus for the initial development of maternal behavior in both dogs and rats.

One clear demonstration of the importance of pup stimuli in the modulation of maternal behavior in dogs is the investigation of the influence of exteroceptive stimuli on epimeletic vomiting. Korda (122) first demonstrated that the main stimuli eliciting epimeletic disgorging of food, an important type of nurturing behavior, is the presence of the puppies close to the mother in the nest-pen, and their care-soliciting behaviors, such as following the mother dog, jumping upon her, and licking her mouth. When puppies were removed from the nest-pen, epimeletic vomiting stopped either immediately or after a few days in 4 out of the 5 observed mongrel mother dogs. Korda (121) followed up this study and carefully examined the *behavioral mechanisms* involved in the process of epimeletic vomiting. He showed that adequate exteroceptive stimuli (nest-pen and puppies) are necessary to elicit epimeletic vomiting. Alterations of such exteroceptive stimuli by either delaying the contact of sated mother dogs with their puppies, changing the place at which the sated mother dogs contacted their puppies, or substituting the puppies of the sated mother dogs with alien hungry puppies disrupted vomiting. One area for future research is to examine how other sensory cues, such as visual and auditory cues, regulate dog maternal behavior. Such research has been extensively studied in rats (65), but not in dogs.

### 7.2 Maternal behavior is observed in adult male and virgin female dogs, as well as in juveniles

Alloparental behavior is quite common in dogs, often observed among a group of dogs who are genetically related and live in a free-ranging condition (15, 16). Free-ranging lactating females are observed to nurse puppies from other kin-related females, and defend each other’s young (16). Male dogs are also observed to play and protect puppies, contributing to almost 70% of active male caregiving behaviors during the observation period. Surprisingly, the overall level of active care provided by the putative fathers is found to be comparable to that of the mothers (mostly nursing and licking behaviors), and much higher than that provided by related non-mother females (15). Interestingly, in dogs, there is a clear labor division in parental care: mothers invest more effort in nursing and allogrooming, while the putative fathers play and protect more. This labor division resembles what has been seen in human parental care across different cultures (145). Juvenile dogs (6 months to 1 year old), especially the old sisters of the young in a social group, can display maternal caregiving behaviors, such as grooming and playing (15). Alloparental care in dogs might be associated with elevated levels of prolactin and oxytocin and lowered levels of testosterone and glucocorticoids in (146).

Because research in this area is often conducted in free-ranging dogs, it is difficult to assess when alloparental behavior begins. In order to do that, maternal behavior could be observed and tested in non-mother dogs (e.g., adult male or virgin females) in the home settings (117). At this point, no such a study has been conducted, thus, whether non-mothers are spontaneously maternal and whether they will display caregiving behaviors towards puppies that are not genetically related to them is not known. We also do not know whether there are two opposing motivational processes (approach and withdrawal) similar to those seen in rats that govern the development of caregiving behaviors in dogs. There is a hint that at least in male dogs, a withdrawal motivational system does exist. Male dogs do not show epimeletic vomiting until they are cohoused with puppies for several days (121). This period might be necessary to suppress the initial withdrawal motivation and allow pup stimuli to activate the approach motivation. Future research could test the validity of this speculation.

### 7.3 Maternal experience effect in dogs has not been established

In rats, it is well documented that interaction with pups induces maternal memory that maintains a long-term enhanced level of maternal responsiveness (59, 147, 148). However, there is little research on the effects of maternal experience in dogs except the above mentioned study on the parity effect (140). Even in that study, it is not known whether maternal experience enhances maternal responsiveness. The case study on grandmother dogs voluntarily taking care of grandpuppies implies that past maternal experience could play a role in the maintenance of maternal behavior in dogs (17). To directly answer this question, we need to know the time course of the development of dog maternal behavior of experienced and non-experienced dogs (e.g., males and virgin females) and identify any difference in the speed of their onset of maternal behavior. We could also utilize the behavioral test paradigms developed in rats to manipulate the amount of experience with puppies at the initial exposure phase immediately after parturition, then test maternal responsiveness at a later re-exposure phase (59, 74). An experience-dependent enhancement of maternal responsiveness should be expected if interactions with puppies do enhance maternal responsiveness in a “dose-dependent” fashion.

### 7.4 The approach vs. withdrawal antagonistic motivational systems have not been demonstrated in dogs

No study has examined whether there are two opposing motivational processes (approach and withdrawal) like those observed in rats that also govern the onset, maintenance and decline of maternal caregiving behaviors in dogs. This represents a clear gap in the literature. The first step to address this issue is to find out whether there is a withdrawal system that opposes the approach system. Theoretically, this could be done by using the “pup sensitization” procedure developed in rats (70, 149). In a typical procedure, 3 to 6 freshly fed donor pups are placed in the home cage of a virgin female daily for a few hours (short enough to not cause any starvation), and its pup-directed responses are recorded, including behaviors indicative of the initial avoidance/withdrawal motivation (e.g., staying away from pups, pushing beddings towards them, attacking them, or shifting nest site, etc.), and those indicative of the approach motivation (e.g., sniffing pups, pushing them, mouthing them, etc.) (102). This procedure is repeated until the female exhibits a clear sign of maternal behavior, showing pup retrieval, pup licking and nursing, etc. or until a certain number of days has passed. The existence of the withdrawal system in dogs is demonstrated when a nonmother dog displays avoidance and withdrawal responses towards donor puppies and needs some time to exhibit full maternal behavior (i.e., no spontaneous maternal behavior). Practically, it might need a strong ethical justification to expose puppies to unknown female dogs for days, even just a few hours a day.

### 7.5 The neural basis of dog maternal behavior is virtually unknown

Similarities and differences in the specific neural mechanisms involved in the control of maternal behavior between dogs and rats are unknown because no study has examined the brain mechanisms of dog maternal behavior. As mentioned above, one of the advantages of studying dog maternal behavior is that various brain imaging techniques (e.g., PET, fMRI, etc.) can be employed to reveal the neural networks and neurochemicals involved in the regulation of maternal related psychological processes (e.g., sensory processing, social and emotional processing, attention, etc.) (150). This remains to be a fertile area for future research. For example, the brain imaging techniques can be combined with pharmacological tools to examine how various monoamines (e.g., dopamine and serotonin) and hormones systems are involved in the regulation of maternal behavior in dogs. Additionally, postmortem canine brain tissues can be carefully examined to identify maternally relevant brain areas.

## 8 Conclusion

I have argued that research on dog maternal behavior has scientific, clinical and practical significance. I also examined the advantages of this line of research over work on laboratory rats and humans. After summarizing the key findings from decades of research on rat maternal behavior and limited work on dog maternal behavior, I discussed the commonalities and differences between dog and rat maternal behavior and point out the deficient areas for future research. For example, sensory, hormonal, and neural controls of dog maternal behavior need to be investigated systematically. Additionally, individual and breed differences could be examined in order to identify the genetic, developmental, social and human factors that contribute to these differences. The motivational control of dog maternal behavior also needs to be elucidated and the relevant neurocircuits need to be identified and compared to those of rats and humans. Overall, because there is dearth of research on dog maternal behavior, much work could be done to enhance our understanding of the common behavioral and neural mechanisms underlying maternal behavior across species.

## Author contributions

ML: Conceptualization, Data curation, Formal analysis, Funding acquisition, Investigation, Methodology, Project administration, Resources, Software, Supervision, Validation, Visualization, Writing – original draft, Writing – review & editing.
